# Medicine and Surgery One Inductive Science; Being an Attempt to Improve Its Study and Practice on a Plan in Close Alliance with Inductive Philosophy, and Offering, as First-Fruits, the Law of Inflammation; Addressed Particularly to the Medical Student and the Profession, but Easy and Intelligible to the Public Also: The Whole Being the Introduction and First Part of a System of Surgery

**Published:** 1838-07

**Authors:** 


### ART. VII.

*Medicine and Surgery one Inductive Science; being an Attempt to improve its Study and Practice on a Plan in close Alliance with Inductive Philosophy, and offering, as First-fruits, the Law of Inflammation; addressed particularly to the Medical Student and the Profession, but easy and intelligible to the Public also: the whole being the Introduction and first Part of a System of Surgery.* By GEORGE MACILWAIN, Fellow of the Royal Med. and Chir. Society; Surgeon to the Finsbury Dispensary; Consulting Surgeon to the St. Ann's Society, &c.—*London,* 1838. 8vo. pp. 551.

WE are not without hope that the exposition which we gave in our last Number of the objects and means of physiological enquiry may have, in a certain degree, answered the purpose which we had in view, by imparting to some of our readers sounder and more definite views than they before possessed of the character of the science, its connexion with other branches of research, and the best means of increasing its comprehensiveness and certainty. It was our intention to follow up the plan which we had there commenced, by laying before our readers, at the first convenient opportunity, a corresponding sketch of the position and prospects of some other departments of medical knowledge, on which it seems to us that similar ignorance and misapprehension prevail. Such an occasion has been presented to us by the publication of the volume of whose title-page we have given a faithful transcript above.

We have often had occasion to remark on the suspicious indications of a voluminous title-page; and we think the volume before us tends to confirm our judgment in such matters. We at once admit that the work contains a good deal of what is both correct and valuable, yet none of this appears to us to possess such claims to novelty as to demand publication in its present bulky and expensive form; still less to entitle the author to assume the credit of having founded a new system of medical philosophy, a distinction at which he evidently aims. We are quite ready to concede to him the disinterestedness of the motives which he sets before us in his preface, (p. vii.;) but, although not writing for present reputation or profit, Mr. Macilwain evidently looks to posterity for his reward. In this expectation, indeed, he does but imitate the Father of the inductive philosophy, whose pathetic appeal to future generations has been so nobly answered; but it seems to us that Mr. M., although full of the language of his great prototype, possesses too little of his spirit to stand much chance of compensation of any kind, for the trouble he has bestowed upon "the medical student, the profession, and the public also."

In noticing a former work by the same author, we bestowed upon him some friendly hints respecting the turgidity and diffuseness of his style: it does not appear, however, that they have produced the effect which we kindly intended, for the volume before us displays the same faults in an undiminished degree. Mr. M. will perhaps excuse himself on the score of his desire to render the results of his reflection intelligible to minds of ordinary cultivation; but we would assure him that plainness and simplicity are qualities more highly prized by that discerning portion of the public to which he addresses himself, than far-fetched illustrations or laboured attempts at profundity, especially when abstract truths are to be communicated and explained. In many parts of the volume, the author has adopted the familiarity of the colloquial style, with the idea, we presume, of engaging and maintaining the attention of his younger readers; but this is a dangerous experiment for a writer of Mr. M.'s powers; since it leads to the diffusion of a vast quantity of common-place over such an extent of surface, that the novel ideas, where they do occur, seem like scattered stars vainly attempting to shine through an atmosphere of mist, which not only obstructs but disperses their rays. In the perusal of Bacon's works, the reader is no less struck with the "boldness of his style, which unites the most sublime images with the most rigorous precision," than enlightened by the profound philosophy of the ideas so felicitously conveyed; and we venture, therefore, to recommend to Mr. M. that study of his great model which he loses no opportunity of enjoining upon his readers. In many parts of his book, Mr. M. shows us that he can write so plainly and unaffectedly that we are led to feel additional regret that he should not always do so. In the following passage from his preface, for example, we perceive not only the result of sound reasoning, but clearness and simplicity in its expression.

"In common with many others of the profession, I have long been dissatisfied with the condition of the science in the cultivation of which the greater part of my life has been employed. I have sensibly felt the humiliating conviction that medical science has not kept pace with other departments of knowledge, either as to its progress or diffusion; and that, as a consequence of this, it is encumbered by many circumstances which impede its progress, abridge its utility, and derogate from the rank of those who follow it as a profession. We can in no way explain these circumstances by any reference either to the paucity of our facts (for of these we have abundance), or to any deficiency of industry in at least a considerable number of the cultivators of medical science; but it is easy to perceive that there is a great difference in the manner which has characterized our investigations, when compared with that observable in the cultivation of the other sciences. In these, there has been a pervading observance of inductive reasoning, as taught us by Lord Bacon: in medical science, we observe as pervading a violation of it. I speak generally: I do not mean that it is always observed in one case, nor always disregarded in the other. When we connect with the foregoing the fact that there is, besides, a very demonstrable correspondence in the progress of every science, and the observance of inductive reasoning in its cultivation, it is impossible to be otherwise than anxious to enquire, at least, whether the slow progress of medical science may not be attributable to that general disregard or violation of rules of reasoning which have so materially assisted the progress of all others." . . . "It has been usual to attribute the low state of medical science to the difficulties attending its cultivation. I can in no way subscribe to this opinion. Like other sciences, it has its difficulties, both general and peculiar, doubtless; but it has also great advantages. The difficulties of which we complain, as resulting from the interminable variety of form under which disease obliges us to investigate the laws of nature, show how little we have wrought in the right way. Properly regarded, these varieties are of the highest utility: they are the strongest and most necessary tests of any law concerning which it may be our object to enquire; and furnish ready to our hands, in countless forms of disease, variations of conditions, which, in other sciences, we are obliged artificially to institute. Besides which, it is only through the multiform *gradations* in disease that we learn the true relations of this 'numerous and dissimilar family;' whilst the very diversity of character which they present produce this remarkable result, that those characters which they have in common (in all matters the most essential to know,) stand out in stronger relief, from the very diversities by which they are in different examples accompanied." (*Preface*, pp. v.-vii.)

Our readers will have no difficulty in tracing the correspondence between the views here expressed and those contained in the first article of our last Number. We there took some pains to contrast the present condition and prospects of physiological science with those of other departments of knowledge, and to analyse the causes which have operated to retard the advance of the former, and to give it a character of uncertainty. When we saw that a similar analysis of medical science was entered upon by Mr. Macilwain, with views which appeared so just, we hoped to be able to continue our former plan, by presenting to our readers the substance of his argument. We must confess, however, that his mode of conducting the enquiry differs so much from the line which we had previously marked out, that we can avail ourselves but little of his labours; and the results appear to us so vague and unsatisfactory, that we scarcely deem it worth while to encumber ourselves with them.

The title of Mr. Macilwain's book, comprehensive as it is, is likely to give rather an incorrect idea of the nature of its subjects. Our readers would scarcely have supposed that, out of the 551 pages which are contained in a treatise headed "Medicine and Surgery one Inductive Science," less than seventy are devoted to its professed object; the remainder containing discussions on the Vital Principle, Sympathy (which subject occupies about 120 pages), and not only the Law of Inflammation promised in the title-page, but the whole theory and practice relating to that disease and its various modes of action. On the present occasion we can only notice, at any length, the subject of the first discourse; but we cannot pass over the remainder of the volume without a few words of criticism.

The second discourse, on Life, displays our author's faults, both in reasoning and style, in a very remarkable degree. The subject is one which peculiarly tests the powers of a writer; and those who wish to compare the best and the worst disquisitions upon it that it has been our fortune to meet with, may place Mr. Macilwain's by the side of that of Dr. Fletcher. The first is as vague and unmeaning as the second is terse and pointed; the former is so full of irrelevant illustrations and laboured attempts at profundity, that whatever reasoning it contains is quite buried beneath the superincumbent rubbish; whilst in the latter there is scarcely a sentence which has not a simple and direct bearing upon the question, and not a single illustration but what is to the purpose. Perhaps the following passage may be within the comprehension of some of our readers: for ourselves we do not profess to understand it.

"The indisposition of the particles of matter to attraction at certain distances is very remarkable, and may be illustrated by a form of matter with which we are most familiar. Even steam will not resume the attractive force necessary to form water, unless heat is abstracted from it; but the tendency to form new combinations is best exemplified by water when it is decomposed. If, for example, we take oxygen and hydrogen, in the proportions in which they form water, we may conceive that, if we mixed them together in a confined vessel, they would combine; but we do not find that this is the case: *they have lost something or other on which their capacity for union depended;* and, in order to restore this capacity, we must subject them to some other influence. In their present state they present phenomena remarkably different from those afforded by water. Instead of being a ready agent for extinguishing fire, they are a most explosive mixture. We find, however, that, if we apply flame, a piece of spongy platinum, or electricity in its more cognizable form, the particles again unite, and water is produced; so that, by separating the elements of water from each other, we have deprived them of the power of reuniting; *and, whatever that is, it is evidently the principle by which the particles of water were held in combination. Deprive water of this principle, it no longer exists as water; its life, as it were, is destroyed.* Now, as regards our present knowledge, we have already arrived at an interesting peculiarity. It is not meant to be inferred that the principle subtracted must necessarily be the principle of life, but it seems reasonable to infer that it must be a link in approximation to it." (p. 75.)

What a mystification is here of the plain and simple doctrines of chemical affinity! Mr. Macilwain's intention would almost seem to have been to perplex his readers into the belief that the ordinary actions of physical laws are as recondite and difficult of investigation as are the operations of vitality; instead of endeavouring to reduce the latter, by a careful and rigid analysis, to the simplicity of the former.

Of the precision of his reasoning and the closeness of his inductions we will give another example; taking the liberty, as in the preceding extract, to indicate by italics the more remarkable expressions.

"If we consider man, we find that he is at once connected with an infinity of other creations, by relations and resemblances which are very remarkable. In every animal, digestion, respiration, and circulation are very analogous phenomena; in many they differ not from those observable in man, either in principle or effect. In vegetables, we have nutrition, respiration, and circulation; and, in inanimate nature, the component atoms of bodies are subject to laws not less rigid and definite. All matter, whether animal, vegetable, or inanimate, is equally indestructible: *all matter* has a certain disposition to preserve its *life* in the form in which we find it in nature, until acted on by influences external to it, the particles of its masses cohering by mutual attraction: all matter has certain tendencies towards other matter. The result at which the mind arrives from this kind of contemplation is, *that the life of every thing, or at least that principle which immediately governs its phenomena, may be the same.*" (p. 84.)

We make the two quotations which follow, with the view of pointing out to Mr. Macilwain the necessity of taking more pains to ascertain facts and study principles, before he ventures to avail himself of illustrations from sciences with which he may not be familiar.

"Some animals can breathe only in air; some only in water; and a few equally well in both. Amphibia, generally so called, are not here intended; these really breathe only in the air: but the Proteus and *Draco volans* are truly amphibious, having both gills and lungs, and appearing to breathe indifferently in air or water. (p. 83, *note.*)

We would willingly presume that *volans* is here a printer's error for *natans*, and that the designation has been applied by Mr. M. to some new species of perennibranchiate Batracian, or to one of the few already known, such as the Siren, Axolotl, &c. The *Draco volans*, as everybody knows, is a beautiful little lizard, which flutters, by means of its parachute-like appendages (formed by an expansion of the skin over its prolonged ribs), among the branches of tropical forests, in pursuit of its insect prey.

"The powers of assimilation possessed by vegetables are very peculiar. In almost every instance, light is essential to them; by a kind of refined analysis, of which we can form no idea but by its result, they appear to derive from light various kinds of colour. That this is the essential mode in which their colour is produced, we infer from the fact that, if deprived of light, and placed under other circumstances not absolutely prohibitory of their growth, no colour is produced." (p. 80.)

The *facts* stated by Mr. M. are by no means correct; for, not to mention other instances, it is well known that Algæ of various species, which inhabit depths of the ocean so profound that they can receive but an infinitesimally small proportion of light, nevertheless exhibit hues of such brilliancy as to bear comparison with those developed under the uninterrupted influence of the solar rays. But what shall we say of his ignorance of the Newtonian doctrine of colours, or of that modification of it which is required by the undulatory theory of light, in supposing that, by some "kind of refined analysis," plants separate the component elements of that complex substance, and, keeping some of them, turn the rest adrift; just as the components of the atmosphere are separated by their respiration? We quite admit that a man may be a judicious and successful practitioner who knows nothing of one theory or the other; but one who comes forward to instruct the public, and aims at the prominent position sought by Mr. Macilwain, might be expected to take the trouble of ascertaining the possibility of his speculations before holding them forth as established facts.

In the chapters on Sympathy we find the same general doctrines inculcated as those contained in Mr. M.'s former work, on the Unity of the Body. He lays particular stress upon his superiority to other practitioners in attention to the subject of constitutional treatment, (on which question we must remain at issue with him;) and tells us (p. 140) of his success in curing "many patients," whom "the first surgeons in England" have failed to restore. In his history of individual cases, however, we are left much in the dark as to the particular means employed. Thus, we are told that, in one case, "his liver was generally solicited by applications to the bowels." Of the complaint of another we are informed, in general terms, that "it yielded to attention to the alimentary canal and skin." In another instance, we find that "simultaneous appeals were made to the skin and the alimentary canal;" and of a young lady, that Mr. M. had "much trouble with her stomach."—But we must pass on to the subject of our especial consideration.

"Surgery," says Mr. M., "is to be regarded as a branch of natural philosophy: it has well-marked and interesting relations to most of the other departments of science, and more or less of connexion with them all." "The idea that surgery is a sort of abstract science," he subsequently remarks, "is false, and therefore injurious. So far is it from being so, that there is no one which it would be more impossible to isolate from general philosophy. In no one branch of human knowledge do we find evidences of that connexion which exists between it and all others more obvious, more intelligible, or indeed more striking, than in surgery." The occurrence of such passages as these, and others which we shall presently quote, at the outset of the enquiry, leads us to enter a little more fully than we should otherwise have thought necessary into the precise character of its objects; for the nature of Mr. M.'s errors (as we, at least, deem them,) too clearly exhibits the vagueness of the ideas prevalent respecting them. We fully agree with him in the belief that, "the more we extend our views into the various departments of natural philosophy, the more are we struck with their mutual connexion;" but this connexion is but superficially and unsatisfactorily perceived until the highest and most general of the laws of the respective sciences are unveiled. To use an oft-repeated but illustrative simily, the branches of a tree may, in their endless ramifications, appear to interlace and unite; but it is not until we trace them back to their common trunk that we perceive their fundamental relation. We do not, therefore, regard the mere dependence of the actions of life upon physical agents, or the cooperation of physical laws in those actions, as indicating this fundamental relation between the sciences of life and those of inorganic matter, which must be determined by the approach of their higher generalizations. All the sciences of life may, and indeed must be, regarded by the philosophical enquirer as closely and fundamentally united; but they are separated by a distinct line from those of inert matter, by the cooperation in them of a set of laws to which nothing analogous is found in the inorganic world. We must, therefore, decidedly object to the classification proposed by Mr. Macilwain, unless the term natural philosophy be regarded as much more inclusive than it is commonly meant to be. We cannot allow that our view of the matter "precludes any enlarged enquiry into the laws, of which diseases are but the exemplification, and debars the science from that light which would otherwise be imparted to it by thousands of philosophically minded men, who are deterred at present from venturing on subjects hitherto represented to them as appertaining to a specific study." (p. 3.) The study of life, whether in the healthy or diseased state, necessarily involves that of its external conditions; and, so far as these are physical, they come within the province alike of the biologist and the natural philosopher. No reasoning founded upon these alone, and proceeding upon the ordinary laws of physics, can go far in explaining the phenomena of life; and these "thousands of philosophically minded men" must be content, therefore, to keep within their especial province, or to commence the study of vital laws in the peculiar method which they require, and on which we have already (in the article referred to) sufficiently enlarged.

But in what sense, it is to be asked, can surgery be legitimately denominated a science? How far can medicine even lay claim to this designation? What are their objects, what their respective limits? We think that we shall make it evident that the consideration of such questions is more practically important than would at first sight appear, since the whole method of our pursuit of these branches of knowledge is involved in it.

"The difference between an art and a science," says Mr. M., "seems to be essentially this: an art consists in the knowledge of certain means by which we can produce certain effects, but without our understanding the principles on which they depend, or, in other words, the laws of nature, of which they are the necessary exemplifications.

A science consists in the knowledge of a certain number of phenomena, and the power of referring such phenomena to the laws by which they are governed." (p. 7.)

Now, this definition is true as far as it goes; but it is by no means the whole truth. It is quite correct to say that, in proportion as a collection of facts is found to be amenable to certain general laws, the attainment of which enables us to extend our knowledge of similar facts, so is the *science* developed in its abstract form. But we hold it to be wrong to maintain that a perfect *art* is anything but the application of a science to practical purposes. It is very true that the *artist* may be himself ignorant of the principles upon which he works; but these, to be of *universal* application, must be deduced from laws of high generality, by a mind fully capable of perceiving their bearings. When this is accomplished, we find that an art assumes a character for perfection exactly proportional to that of the science upon which it is founded, after due allowance is made for the uncertainty resulting from the intervention of human agency.

We formerly spoke of astronomy as the most perfect of the sciences, since all its phenomena can be reduced to one general principle. Its most important practical application is the *art** of navigation, which is a collection of rules framed by those profoundly conversant with the principles of the science, but capable of being employed by those whose knowledge extends no further than the mode of applying them. It is well known that, to such perfection has the art of navigation arrived, that it is quite possible for the place of a vessel to be determined within a mile, after she has been driven by winds and currents for many months subsequently to the last accurate knowledge of her position. For such precision two kinds of conditions are evidently requisite: the certainty of the rules, arising from the impossibility of their correctness being impaired by the intervention of any unknown cause, which can only be secured by the comprehensiveness of the principles on which they are founded; and the skill of the individual concerned in the application of these rules, the *artist*, and the perfection of the instruments which he employs. These conditions are obviously so independent that a profound mathematician, capable of admirably fulfilling the first, may be totally inefficient with regard to the second; and, in like manner, a correct observer may easily acquire the knowledge sufficient to avail himself fully of the rules of the art, although totally ignorant of the abstract principles on which they are founded.

We have purposely taken this illustration from an extreme case, that we might be able to enforce our doctrines with more clearness; but we shall find the results to be similar if we turn to other sciences much less advanced, and enquire into the state of some of their dependent arts.

In chemistry, as we have already stated, the facility of experimental enquiry has led to the establishment of laws of a high degree of generality; but these require so much qualification and restraint in their application to particular instances, that it is hazardous to predict unknown phenomena, especially where organic compounds (of whose nature we are more particularly ignorant) are concerned. Hence no art

* Navigation may also be regarded as a *science;* but it is then only a branch of the science of astronomy.

founded upon this science can be supplied with other than very limited rules. In the case of dyeing, for example, great improvement has been effected by chemical knowledge; but the greater part of its rules are *empirical*, that is to say, founded upon a limited induction, not comprehended in more general principles, and therefore quite uncertain in their application to unknown cases. Thus, if a new animal or vegetable dye were discovered, the modes of fixing and discharging it, and of varying its shades of colour, would have to be determined by experiment before it could be brought into advantageous employment. We can, nevertheless, imagine, and in some degree anticipate, the period when chemical science shall be so far advanced that a simple analysis of the material (analogous to the *observations* of the navigator) may enable the manufacturer, by a reference to his code of rules, to avail himself to the fullest extent of its capabilities, without being himself aware of the principles upon which those rules are founded. As long as science and art are in a progressive state, it is obviously advantageous that the *artist* should be cognizant of the principles of the science, that he may not neglect opportunities for the improvement of both; but, when once perfected, we have seen that no such utility exists.

An art, then, will be *scientific* or *empirical** according to the comprehensiveness of the principles upon which its rules are founded. A little consideration will show that this distinction has nothing to do with the certainty or uncertainty of its applications in particular instances. An art may be entirely empirical, and yet be perfect *so far as it goes*; but no unknown cases are provided for, no contingencies foreseen. It is in their adaptation to these that the triumph of a scientific over an empirical art manifests itself; and, in proportion as, from the nature of the subjects embraced by it, a greater or less variety of novel cases presents itself, in that proportion is the superiority more evident. The deficiency of higher or more comprehensive laws should not prevent us in any instance from making cautious use of those we already possess; and, where the demands of mankind require that an art should be practised even in its imperfect condition, we must be content with such means of satisfying them as lie within our reach. Contentment, however, by no means involves a tacit acquiescence in the infirmities of our condition; and the man of noble and elevated mind will not only aim at the perfection of his science from that abstract love of knowledge, which, as Sir H. Davy has beautifully observed,† "is, in fact, in its ultimate and most perfect development, the love of infinite wisdom and unbounded power, or the love of God," but may also safely cherish the belief that every contribution he makes to the establishment of general laws will ultimately have its practical bearing upon the condition of humanity, and that future generations, if not his own, will be benefited by it. It was beautifully observed by Lord Bacon, that "it is the office and excellence of all sciences to shorten the long turnings and windings of experience;" and we shall hereafter see how forcibly this remark applies to the topics of our present consideration.

We return to Mr. Macilwain's attempt to rank surgery as a science:

* See Herschel's Prelim. Disc.; p. 70, &c.

† Consolations in Travel; 3d edit., p. 53.

"Medical science," he says, "is, in fact, the study of the laws and relations of animal bodies, in order to ascertain the modes in which nature relieves diseases or repairs accidents; and to determine the conditions of the whole body which favour or impede these processes in its various parts; with an especial view, in the one case, to the *maintenance* of such conditions; and in the other, to their *removal.* The achievement of this point is the common object of medicine and surgery. Surgery is sometimes called an art. If it be an art, however, it is one of the lowest description; inferior indeed to the most ordinary handicraft. As well might you call astronomy an art, because it requires the adjustment of optical instruments. Surgery is never an art but in consequence of its imperfection as a science; and even then it is one of very simple character. As an art, and one that can be acquired in less time than any with which I am acquainted, it will remove a stone from the bladder, will tie an artery, or will amputate a limb; but, as a science, it would prevent the formation of the stone, the changes in the coats of an artery, or the forces of the circulation which produced the aneurism, and put a stop to the morbid actions necessitating the removal of the affected member." . . . "In thus contending that surgery should be regarded as a science, it is necessary to observe that I speak more with reference to what it must necessarily be as a branch of natural knowledge, than to any *actual* possession which we have of such knowledge." (pp. 6, 7.)

In the foregoing observations, Mr. M. seems to us to have laboured under some difficulty, through confounding the two senses in which the term art is commonly employed. In many cases it is used synonymously with "skill, dexterity, or the power of performing certain actions acquired by experience, study, or observation." In this sense, it would be correct to say that the mere performance of many surgical operations requires art of the lowest description. We have seen many bones sawn through in a manner that would disgrace the most ordinary workman; and the lithotomic skill of Frère Jacques might easily have been acquired by a barber or butcher properly instructed in his method.*

But art, in its higher sense, implies "a system of rules serving to facilitate the performance of certain actions;" or, in the words of Sir J. Herschel, (the worthiest representative of Bacon in our time,) it is "the application of knowledge to a practical end." It is evident, therefore, that, as the essence of medicine and surgery consists in their practical applications, they are to be regarded as arts and not *sciences;* and it is to be lamented that their rules are as yet so little founded upon general principles, and stand so frequently upon the narrow basis of a limited induction, that we must consider them as rather empirical than scientific.

We formerly showed† that the changes which characterize living beings, and which in their totality constitute their life, are capable of being referred to certain general laws expressive of their uniform conditions. Organized structures possessed of vital properties, on the one hand, and the elements of the inorganic world on the other, afford these conditions; and, by the study of all the phenomena which are presented by the actions thus resulting, the science of physiology will, we doubt not, be gradually built up and perfected. To pursue it with advantage, an acquaintance is necessary with the characters of the conditions themselves; and thus arises the study of organized structures on the one hand, and that of much of this difficulty, however, to the backward state of the science of

* We believe it to be a fact pretty generally known, that after this celebrated empiric was made acquainted with the anatomy of the parts which his knife incised, he was much less successful than before, in consequence of his more timid method of operating.

† British and Foreign Medical Review, vol. V. p. 328, et seq.

general physics on the other. Anatomy is not a science as long as it is a mere collection of facts, "rudis, indigestaque moles," but as soon as the laws regulating the structure and development of the countless varieties of living beings begin to be evolved, the chaos of materials assumes a definite order; and one of the most beautiful as well as most interesting —because most rapidly advancing—of all sciences, philosophical anatomy, presents itself.

Physiology, then, being the science of *normal life*, if we may be permitted the expression, it will be enquired what is its practical application,—to what *art* does it furnish rules? The answer is easy: to the art of preserving the body in health. How successfully this has been accomplished of late years, and how rapidly the diffusion of sound physiological principles is spreading more rational ideas as to the education and management of the body and mind, we need not stop to point out. As physiology is still, however, but a very imperfect science, the rules of art deduced from its principles are, like those of chemistry, limited in their application, and not trustworthy in novel and untried instances. We do not anticipate the return of an epoch of patriarchal longevity, whatever may be the ultimate perfection of physiology as a science; but the experience of the last century has amply shown that attention to its simple and universally acknowledged truths will go far towards both the prolongation of life and, a not less important object, its emancipation from disease.

The slightest observation of the actions performed by living beings proves to us that, independently of those *secular* changes which cause the decay of individuals and races as constantly as their renewal, there are occasional deviations from this regularity, which constitute the phenomena of disease. The investigation of these phenomena, and the reduction of them to general laws, expressive of their conditions, is the object of pathology, the science of *abnormal life*. Here, as in the kindred science of physiology, the study of all the conditions is requisite; and hence we have to make ourselves acquainted, on the one hand, with the characters of all the external agents which can produce a deleterious effect upon the living body, whether their operation be mechanical, chemical, or vital; as well as with the results of the suspension, partial or complete, of the conditions by which its healthy action is maintained. On the other side, we have to investigate the changes of structure which manifest themselves in the body itself; and the countless variety of secondary results which arise from any disturbance of its regular train of actions. The difficulties which beset us in these enquiries are of a corresponding nature to those which we have set forth as encumbering the pursuit of physiology. We shall be comparatively brief, therefore, in the exposition of them.

The pathologist sets out with endeavouring to determine the individual phenomena of diseased action; and, when he has collected these in sufficient amount to serve as the basis of an induction, he endeavours to determine the conditions common to all, and hence to arrive at their laws. With what difficulties the first part of the investigation is encompassed is sufficiently evident from the discrepancy of opinion which prevails as to the nature of the simplest and most easily observed changes; such as those which occur in inflammation. We cannot but attribute physiology. Until the normal functions of the different organs are thoroughly understood, we cannot deem it probable that there will be much consent as to the character of the deviations from them; and, in the case we have referred to, it seems to us necessary that physiologists should agree upon the connexion between the motion of the blood in the capillaries, and the vital properties of the fluid and the changes it ought to undergo in them, before a correct statement can be made of the morbid phenomena of inflammation. Such a statement would not be entitled to rank as a *law;* it would only be a *fact*, as we formerly showed with regard to the circulation, &c.; but, if the nature of changes which can be brought under direct observation is likely to remain for some time a disputed topic, how much more difficult must it be to discover those which occur in the recesses of the system! Great as have been the improvements in diagnosis of late years, they too often only reveal to us the sensible *results* of the changes which constitute diseased action, rather than the original changes themselves; and, in reasoning backwards to the cause, we are obviously exposed to fallacy at every step, from the limited use of general principles to which our ignorance of vital actions restrains us. Thus, auscultation will reveal to us the deposition of tubercular matter in the lungs, and the *consequent* chain of phenomena; but it will give us no information of the character of the *previous* change in the capillary circulation which is the cause of the deposit; and our knowledge of it is therefore still more indefinite than of that concerned in inflammation.

It is evident, therefore, that the difficulties of diagnosis are not only felt by the practitioner (as they long must be,) who endeavours to make his scientific knowledge available for the exercise of his art; but they also obstruct the progress of those researches into the elementary phenomena of disease, upon which alone the general principles of pathology can be established. The observation of the morbid changes produced by disease, either when presented to our inspection during life, or discovered by examination after death, may, in some degree, supply the deficiency; but this too often supplies us with information of no higher character than that derived through other channels, revealing to us only the *results* of diseased actions, rather than the nature of the actions themselves.

But the difficulties are greatly enhanced when we consider that the cases of abnormal action, in which the structure of the part functionally deranged undergoes a sensible alteration, bear but an extremely small proportion to the instances of sympathetic disorder from irregularity of some other portions of the series of vital changes. We have said of nervous agency in the healthy state, that we know nothing of its nature, and can only judge of it by its effects; the same observation may be applied with increased force to the state of disease; for it would seem useless to hope for the detection of any physical changes in the nervous system corresponding with the results which disordered innervation produces through other organs: and that which must be kept in view in reasoning on morbid phenomena in general is especially applicable to the nervous system, that we must be careful not to connect too hastily the observed symptoms with the morbid appearances. From error in this respect many erroneous doctrines have arisen. Our object should rather be to discover what has been the primary morbid action of which the diseased condition is the sequence, and whether a similar morbid action may not have occurred in cases where we find no such *evident* result.* Thus, epilepsy is a manifestation of abnormal action of the nervous centres, of the nature of which we are profoundly ignorant: this abnormal action may originate in disorder of a distant organ, and may leave no recognizable manifestation after death; or it may be the result of some other change in the organs themselves, which, leaving manifest traces, may be hastily set down as the proximate cause of the disease; or, from its long continuance, it may produce some structural alteration, which may in like manner be hastily conceived to be the cause of the symptoms when it and the symptoms are the common result of some still mysterious cause.

The difficulties which impede our attempts to become acquainted with the changes in the external elements, and the operation of deleterious agents are sufficiently great to retard the progress of the enquiry; especially whilst we are still so much in the dark respecting the natural effects of vital stimuli. We cannot, however, regard them as by any means insurmountable; and, though we cannot weigh or measure the quantity of malaria in the atmosphere or bring into a tangible form, the fomites of a contagion, we may make ourselves acquainted with the laws of their origin and distribution, and the mode of their deleterious action on the body. We think it will appear, then, that, by a judicious subdivision of labour, the science of pathology may gradually be elevated from its present uncertain condition; and that, by the cautious generalization of carefully-sifted and correctly-observed facts, abstract principles may be developed, possessing a certainty not inferior to those of the so-called *exact* sciences. As we formerly observed, the process of induction is essentially the same in all instances. It is in the establishment of individual facts that the great difficulties exist, which have so much retarded, and will continue to delay, the progress of the sciences of life. We should recollect, however, that the infirmities of mankind are constantly presenting us with the opportunities for observation; and that the variety of diseased phenomena may be rendered, as Mr. Macilwain has justly remarked, a source of information, rather than of perplexity. It is the great dissimilarity of the facts so collected which prevents any one but a sagacious philosopher from discovering the common tie which connects them all. The brilliancy of Newton's genius was shown in the perception that the fall of a stone to the earth and the motion of the moon around it were analogous phenomena, subject to the same law; not in the mere deduction of the numerical law from the ratios supplied by these facts.

As the *art of preserving health* arises from the proper application of the *science of physiology*, so does the *art of curing diseases* or injuries depend upon the *science of pathology*. But its rules must result in part from the application of another science, that of therapeutics, which might justly, however, be considered a branch of pathology, or the science of disease. As life, in the healthy condition, is known to be maintained by the action of external agents upon organized tissues endowed with vital properties, so is it found that, in diseased states of the system, such a change takes place in the character of these actions as adapts them to its altered circumstances; and it is also found that, by artificial regulation of the natural actions, or by the substitution of new ones, the diseased

* See British and Foreign Med. Review; vol. IV. p. 428.

condition may frequently be controlled, and a state of corporeal sanity be restored. The enquiry into the curative influence of external agents upon the phenomena of disease is, therefore, a branch of the general science of pathology (though not usually regarded as such,) which corresponds precisely to the study of the ordinary relations between organized structures and the external world, which is universally allowed to be a most important branch of physiology.

The *ars medendi*, then, or the *practice* of the healing art, is based upon the *ratio medendi*, or its *theory*. Were the science of pathology more nearly perfect, the rules deduced from its general principles would be of easy application, and would require only correct observation of the circumstances which called for their employment: but this, unfortunately for us, is very far from being the case. Were we able to devote all our energies to the advancement of pathological philosophy, we might venture to anticipate the commencement of this medical millenium: but the infirmities of mankind continually distract our attention by the claims they put forward for the imperfect assistance which we are as yet able to afford them. Hence we find that the attention of those who devote themselves to the study is directed rather to the alleviation of suffering by the temporary expedients which a limited experience has shown to be useful, than to the establishment of higher and more general principles which might for a time be barren of practical results. From this it follows that medicine, as an *empirical art*, has advanced far beyond the science of pathology; and that it has little claim to be regarded as a *scientific art*. That its rules are based upon an extremely narrow foundation is evident to any one who attempts to extend their application to novel cases, as well as to those who have experienced the distressing uncertainty of medical practice in the inability to control particular forms of disease by the means usually efficacious. It is from the want of analysing the causes of this uncertainty, and from misunderstanding the present position and prospects of medicine, that many ardent and hastily-judging men have abandoned the subject in disgust, under the notion that no sciences connected with the constantly-varying actions of life could ever emerge from such a chaos of ignorance and uncertainty. We trust, however, that we have shown in this and a former article, that the actions of living beings, whether normal or abnormal, are as amenable to general laws as those of inert matter; and that the discovery of these laws is within the reach of those who search after them in the right track. That we are still so far from attaining them is not to be wondered at, when we reflect upon the errors and absurdities of the methods which have been employed in their pursuit: one class of reasoners hastily raising a pile of flimsy principles upon a few ill-observed facts, and calling upon the world to acknowledge their truth, and be killed or cured upon the rules of art derived from them; and another falling into the opposite extreme, and doggedly refusing to call in the aid of reason at all, or to advance one step beyond the bounds of certain experience, however limited. The latter error has been very properly reprobated by Mr. Macilwain.

"In animals, and (from the superaddition of moral causes) especially in man, we have not the same opportunity of assuring ourselves that we have observed phenomena in any two cases under precisely the same circumstances, as we possess in the study of physical changes. No wonder, then, that we cannot as easily arrive at conclusions by strict induction as they can who pursue other sciences; but it is to be feared that the task, when its difficulty should have given rise to a corresponding increase of exertion, has been too often rejected as hopeless; and that, because we could not arrive at conclusions which were certain, we have not sufficiently striven at such approximations as are really within our grasp." (pp. 36, 37.)

Accordingly, in the practice of medicine, our aim must be to avoid, on the one hand, confiding too implicitly in general principles, however stable and comprehensive we may imagine them to be, until we are satisfied that we know not only the principles themselves, but the subordinate laws which regulate or modify their application to individual cases. Long after the highest laws of motion had been established by Newton, no astronomer could, on the faith of them, have predicted the situation of a planet with more than an approximation to certainty: the law of attraction had to be applied in numberless modes not contemplated by its discoverer, before perfect accuracy could be attained. There is great danger, then, in the present state of the science of pathology, in trusting to principles which we may consider unassailable, as our sole guides in the practice of our art; and hence it is that it is not always the *scientific* practitioner, as he is emphatically termed, who is the most successful in his treatment. Nothing, indeed, can more strongly display the present uncertainty of medicine than the superior efficacy often to be witnessed in the treatment of an acute and observant watcher of symptoms, who may not even pretend to form a diagnosis. The other extreme, however, is equally to be avoided, especially by those who aim at something more than individual success. We read in Celsus of the Εμπειρικοι of his day, who professed to confine their practice within the limits of experience only; but, as he very justly remarks, some kind of reasoning is necessary in the application of experience, however acquired, to particular instances. The recorded experience of ages, in its condensed form, must of necessity assume the appearance of general rules of practice: hence, no one, who professes to be guided by it, can avoid making more or less use of scientific principles. The *rational empiricism*, as it has been termed, which prevails in this country at the present time, may justly, we think, be regarded as the most advantageous means which could be pointed out for advancing the progress of pathology as a science, at the same time that use is made of medicine as an art. The value of *facts* as the only sure basis of general principles is duly appreciated; and yet there is no indisposition to make trial of such principles when announced, and to abide by them so far as they shall appear practically available. The *empirics* of the present day, however, whether within or without the pale of the profession, by no means take the ground occupied by those of the ancient schools. Instead of abiding within the narrow limits of experience, they erect the most hasty generalizations upon a few limited instances, and trumpet forth a remedy as endowed with panaceal virtues, or as curing all forms of particular diseases, which has been in some individual cases successful.

Whilst, then, we acknowledge the value of the contributions which have been made (and which we continue to expect) to the science of pathology, by those who apply medicine and surgery as arts, we cannot but believe that great advantage would result from exclusive devotement to the former of men qualified by original character and extended education to labour in its higher departments. We say extended education, because we believe, and we hope to have satisfactorily shown on a former occasion, that no man is so likely to reason philosophically in the investigation of vital phenomena, as one who has received a careful training in the school of physical science. We quite agree with Cullen and M. Louis in the belief that there are false facts as well as false theories in medicine; and that a long course of observation is necessary to establish the basis alone of future inductions. The band of observers, therefore, which the last-named physician is training up *may* contribute much to the advancement of *science;* but we doubt much whether they will do more, on their present plan, than render the *art* more empirical.

We will not do the author the injustice to close this article without noticing so very material a part of his work as that which enounces the *Law of Inflammation,* to the discovery of which Mr. Macilwain believes that he has been led by the application of the inductive process to the phenomena of disease.

"The law, then, is, that inflammation is essentially a reparative process, and that, where the repair required is that of local injury, it is of course referred to the seat of such injury: in all other cases, it is a process instituted by nature to get rid of injurious influences, by determining to the surface of the body." (p. 285.)

To what degree of novelty this doctrine can lay claim in regard to inflammation in particular, we shall not stop to enquire; simply referring our readers to the works of John Hunter, and especially to section sixth of his chapter on the Fundamental Principles of Inflammation, where we meet with the following passage, which has been so often commented on as to be tolerably familiar, in substance at least, to most students of the present day: "Inflammation is to be considered only as a disturbed state of parts, which requires a new but salutary mode of action to restore them to that state wherein a natural mode of action alone is necessary. From such a view of the subject, therefore, inflammation, in itself, is not to be considered as a disease, but as a salutary operation consequent either to some violence or some disease."* Whatever may be the validity of the principle itself, we cannot but feel some surprise that Mr. Macilwain has brought it forward as the result of his philosophic researches, without any reference, that we have met with, to the well-known doctrines of Hunter; and our readers will probably be led to form no very exalted opinion of the powers of reasoning displayed by our author, when they compare this *law,* professedly deduced from the observation of phenomena, with the *postulate* on which he started (p. 95), and which is as follows:

"That the body contains within itself certain powers of preservation, of maintaining an equilibrium under a variety of disturbing influences; *that diseases, in all their diversified forms, are, in fact, but actions of this preservative power;* and that the operation of the latter takes place, subject to certain laws of limitation, of which our present knowledge enables us to form no very correct idea."

To us it appears that the latter (if admissible) is the more general law of the two, and that the former is only a particular instance of it. It is

* Hunter's Works, vol. iii. p. 296.

a maxim based on common sense, as well as on higher authority, that we shall judge of things by their fruits: assuredly, the specimen we have had in the *first*-fruits of Mr. Macilwain's application of inductive philosophy to medicine and surgery, does not encourage us to look for any more valuable ones at a future time.

---

### Art. VIII.

*De la Morve et du Farcin chez l'Homme.* Par P. Rayer, m.d., Médecin de l'Hôpital de la Charité, &c. (*Mémoires de l'Academie de Médecine*, tome vi.)—*Paris*, 1837. 4to. pp. 878.

*On Glanders and Farcy in Man.* By P. Rayer, m.d. &c.

